# Use of Nanocomposites in Bone Regeneration

**DOI:** 10.7759/cureus.31346

**Published:** 2022-11-10

**Authors:** Neha Masne, Ratnakar Ambade, Kunal Bhugaonkar

**Affiliations:** 1 Medical School, Jawaharlal Nehru Medical College, Datta Meghe Institute of Medical Sciences, Wardha, IND; 2 Orthopaedics, Jawaharlal Nehru Medical College, Datta Meghe Institute of Medical Sciences, Wardha, IND

**Keywords:** orthopaedics, nanomedicine, regenerative medicine, tissue engineering, nanocomposites, bone graft

## Abstract

This article examines the compositions of bone tissue grafting and presents tissue culture and engineering formally as an approach for orthopaedic surgery. We assessed articles on bone grafts, analyzed their properties, advantages, and restrictions, and delivered explanations on technologies, including bone-tissue engineering (BTE). Osteo graft materials range from real human bone autografts (self-grafts) to substitute materials that can be used as grafts. These can be used single-handedly or conjointly to improve bone healing and regeneration. Tissue engineering is a relatively newer and evolving alternative for reducing the challenges of bone grafts and improving the rehabilitation of bone fractures and defects. Shortly, the combination of scaffolds, healing factors, gene therapy, and, more recently, 3D printing of tissue-engineered constructs may yield new perceptions. Natural bone tissue has a nanocomposite structure that offers the right biological and physical characteristics. It is essential that the biomaterial resemble real bone tissue in order to regenerate bone tissue. Because they can offer the correct matrix environment, combine desirable biological features, and allow regulated, sequential distribution of numerous growth factors for the different phases of bone tissue regeneration, nanocomposites are the ideal alternative for bone tissue regeneration. This is because no single type of material can replicate the composition, structure, and characteristics of native bone. A relatively new class of materials called nanocomposite biomaterials combines a biopolymeric and biodegradable matrix structure with nanoscale fillers that are bioactive and easily resorbable. There are also some things to think about when using nanoparticles and nanocomposites as scaffolds in clinical settings for bone tissue engineering.

## Introduction and background

Bone is a metabolically active, dynamic and versatile organ with a peculiar regeneration quality. Along with the weight-bearing action and aiding mobility, the 206 total bones in the human body provide a variety of biological functions, including blood cell formation (haematopoiesis), safeguarding the critical organs of the body, including the brain and heart, and mineral and various growth factors storage [1]. Cells, fibrous protein, collagen, hydroxyapatite, and water [2] make up the majority of native bone. It has a nanocomposite structure with physical and biological properties that are acceptable [3]. Bones, unlike other tissues, may regenerate and mend themselves: many bone injuries and fractures recover without scarring [4]. However, the bone healing process can be hampered in cases of bone tumour excision, injuries with severe abnormalities, or infections, preventing the affected area from entirely and spontaneously regenerating [1]. Bone grafts or bone replacements are used in approximately four million surgeries each year [2]. Autografts are still the gold standard for minor defect rehabilitation. However, they come with a slew of disadvantages, including infection, haemorrhage, a finite amount of donor bone tissue, the requirement for another operation site for bone graft harvest, donor site morbidity, and persistent discomfort. Tissue engineering (TE), which strives to repair, maintain, and improve the functioning of injured organs from a reservoir of regenerative cells efficient for both self-renewal and regeneration, offers novel options for the treatment and regeneration of bone tissue; however, if a bone tumour is removed, traumas with significant differentiation in other cell types are likely to occur [1]. TE is defined as a method to alter the structure and architecture of any viable and non-viable tissue with the goal of increasing the construct's effectiveness in biologic environments [4]. The structure, composition, and characteristics of advanced nanocomposites for bone tissue regeneration are discussed in this article. It addresses biomimetic bone-like nanocomposites manufacture, guided bone regeneration employing inert biomaterials and bioactive nanocomposites, and nanocomposite scaffolds for bone tissue regeneration [3].

## Review

Properties of grafts and bone substitutes

Critical-size bone defects (CSDs) are treated with regenerative therapy using bone grafting, and a variety of prosthetic bone materials have been developed in this field. One that does not heal naturally throughout a patient's lifetime and requires surgical stabilisation and additional surgical intervention is what is meant by a defect, according to definition [5]. An ideal bone graft must be biocompatible, biodegradable, osteoconductive, osteoinductive, structurally similar to bone, easy to use, and cost-effective. The ability of osteoblasts to generate new bone is known as osteogenesis. The ability of graft materials to trigger the development of bone-forming cells is known as osteoinduction. Osteoconduction is a feature in which the graft acts as a permanent anchor. Tissue engineering includes all non-fresh grafts that have been processed for acellularization. Only autografts have all of the above characteristics among all forms of bone grafts [4].

Nanomedicine in bone tissue engineering (BTE)

**Figure 1:**
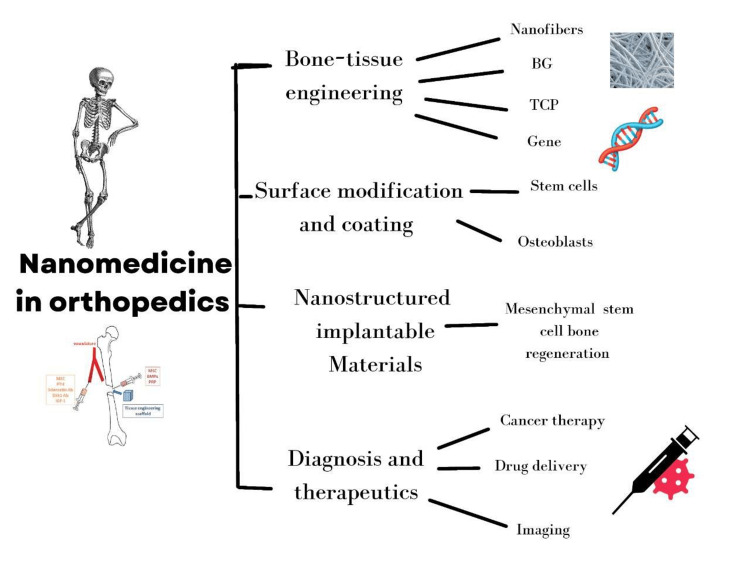
Potential applications of nanomedicine in orthopaedics BG- Bioactive glass; TCP- Tricalcium phosphate Image credit: Author Neha Masne with reference from Mazaheri et al., 2015 [6] (Open Access)

Nanomedicine, or the application of nanotechnology in medicine, tries to solve disease-related problems at the nanoscale, where most biological molecules exist and function [6]. At the nanoscale, polymeric nanofiber networks can imitate the structure of normal human tissue. Because of their high surface-to-volume ratio [7], the networks effectively facilitate cell adhesion, proliferation, migration, and differentiation. According to accepted definitions, polymer nanocomposites are multiphase materials with at least one dimension less than 100 nm. The successful creation of nanocomposites is typically attributed to two major factors: a large specific interfacial area and stress transfer across the interface that is controlled [8]. Nanomaterials are interesting options as future and alternative orthopaedic materials due to their ability to mirror the size of constituent components of normal bone (Figure 1) [6].

**Figure 2:**
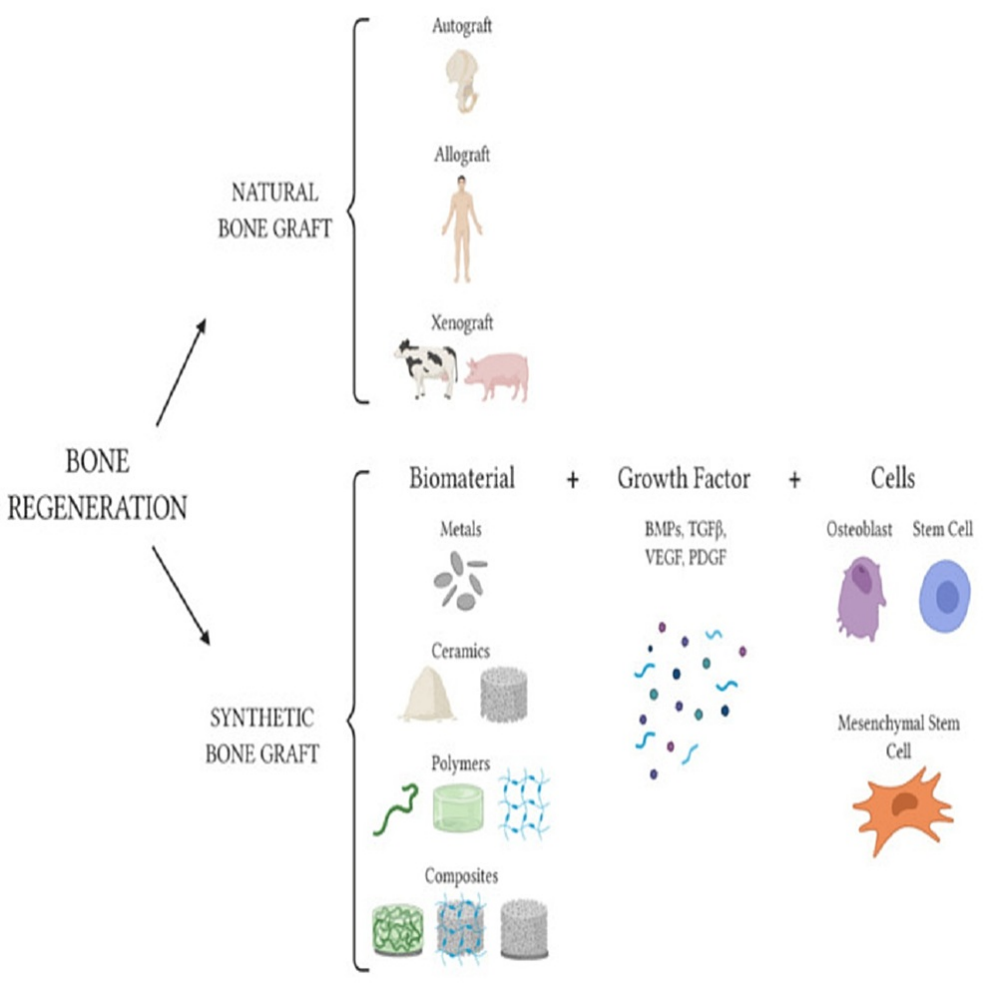
Schematic of available options for bone tissue regeneration Autograft, allograft from human donors, and xenograft from other species are all examples of natural bone transplants. Metals (grey discs), ceramics (beige powder and grey microporous cylinder-shaped item), and polymers (green ribbon, green stiff cylinder-shaped hydrogel, light blue hydrogel mesh) can be used to create synthetic bone grafts. Growth factors such as BMPs (bone morphogenetic proteins; BMP-2, BMP-7, BMP-9 here represented as blue and green circles and violet hexagon), TGF (transforming growth factor-beta; pink hexagon), VEGF (vascular endothelial growth factor; blue ribbon), and PDGF (platelet-derived growth factor; light blue ribbon) can be used to functionalize biomaterials. Furthermore, cells can colonise a synthetic bone transplant. Image source: Battafarano et al., 2021 [10] (Open Access)

Since the advent of the "tissue engineering" idea 25 years ago, strategies for bone tissue regeneration have constantly been changing [9]. Synthetic bone grafts are classed as biomaterials, substances engineered to interact with living systems and intended for tissue replacement, that can be classified depending on their composition in metals, ceramics, polymers and composites (Figure 2) [10].

Nanomaterials in BTE

There are two types of nanomaterials that can be used in this field: organic and inorganic. Polymeric nanofibers are organic nanomaterials that are particularly useful for the production of highly porous scaffolds for cell growth. Inorganic nanoparticles are used as they are or dispersed in matrices to enhance their functional capabilities while maintaining excellent biocompatibility. They come in a variety of shapes (nanoparticles, tubes, and fibres), and when combined with organic matrices, they can improve a variety of qualities (biologic, mechanical, electrical, and/or antimicrobial). Their huge surface-to-volume ratio is another desirable feature. It allows nanoparticles (NPs) to pass through the membrane and aid protein absorption in cells [7]. Several types of biodegradable scaffolds are designed to keep regeneration cells in place, remake their microenvironments, and support new tissue growth until integrated operated region [1]. The results show that all nanocomposites have favourable in vitro cytocompatibility and could be used for improved functionality (Table 1) [11].

**Table 1:** Various nanomaterials, their advantages and disadvantages, and modified nanocomposites available PLA- Polylactic acid; CNT- Carbon nanotubes; PCL- Polycaprolactone; TCP- Tricalcium phosphate; HA- Hydroxyapatite; FA- Fluoroapatite

Nanomaterials	Advantages	Disadvantages	Modified Nanocomposites	References
CNT	High structural stability, Functional group modifiability	Cytotoxicity	(PLA)/CNT composite scaffolds, CNT-coated PCL-PLA scaffolds, Poly-DL-lactide	[2,12,13]
PCL	Bio-absorbable, Biocompatibility, Easy handling, Economical	High degradation, May elicit a strong inflammatory response, Lack of rigidity, Stability	PCL membranes, seeded in mesenchymal cells	[14,15]
Fibrin	Commences hemostasis, Cell adhesion, Serves as matrix	Naturally occurring, Not many structural similarities	Fibrin-chitosan/nano-TCP composites	[16]
Chitosan	Biocompatibility, Biodegradable, Non-protein matrix	Glutaraldehyde toxicity, Not cost-effective	HA-chitosan scaffolds, Chitosan/PCL scaffolds	[8,15]
Fluorapatite	Limited solubility, Osteointegration, Structural compatibility	Similar to HA plasticity	FA-collagen/gelatine/PLA scaffolds	[17]
Calcium Carbonate Crystals	Mirrors structural composition, Improves osteoblast activity	Low toughness	Aragonite-CaCO3 nanocrystals	[15,18]

Biomaterials in bone regeneration

A. Human Stem Cells

Human stem cell research is an intriguing field of study with the potential to significantly impact medicine. Stem cells are an ideal target for manipulation in translational regenerative medicine because of their self-renewal and ability to give rise to multiple cell types. Although regenerative medicine research has the potential to be translated into clinical practice, the biology of haemopoietic pluripotent stem cells (HPSCs) and in vitro-produced cell types, and tissues for the craniomaxillofacial area needs to be better understood before they can be used safely inpatient [19]. The osteogenic development of unrestrained somatic stem cells was increased by porous poly(L-lactic acid) (PLLA)-based scaffolds with hydroxyapatite (HA) coated on the surface of nanofibers. The optimum bone repair scaffold material should deteriorate during the first two months of bone repair. Because of collagen (Coll) degradation, PLLA/Coll scaffolds had a fractured morphology after 80 days [20]. In order to predictably restore the confused architecture and function of bone, functional BTE essentially uses osteoprogenitors obtained from mesenchymal stem cells (MSCs) and seeded onto a scaffold [5]. MSCs produced from embryonic stem cells (ESCs) or induced pluripotent stem cells (iPSCs) may be a new source for BTE studies [21].

B. Naturally Derived Biopolymers

These are naturally obtained biopolymers that are commonly employed in BTE applications; however, these materials have limitations because of their instability, compatibility, immunogenic response, and slow biodegradation [5].

The most widely used naturally derived polymers are Coll-based membranes, which are crucial for cell-matrix communication [22]. Coll membranes' primary drawback is that they lack sufficient stiffness; therefore, their application is more appropriate for conditions like alveolar bone defects [23-25]. The majority of commercially available Coll membranes are made from type I or a mix of type I and type III Coll. Coll is derived from bovine, porcine, or human tendon, dermis, skin, or pericardium. Coll materials have several advantages for use as a barrier membrane, including hemostasis, chemotaxis for periodontal ligament fibroblasts and gingival fibroblasts, low immunogenicity, ease of manipulation and adaptation, a direct effect on bone formation, and the ability to augment tissue thickness. As a result, Coll looks to be an excellent alternative for a bioresorbable BTE barrier. A type I highly cross-linked Coll membrane was shown to be associated with a virtually full continuous layer of lamellar bone with osteoblastic activity after 30 days in a rabbit study by Colangelo et al., compared to solely fibrous connective tissue in the non-membrane control group [24].

Chitosan (CHI) is a semicrystalline linear polysaccharide derived from the deacetylation of chitin and consisting of N-acetyl D-glucosamine and D-glucosamine units joined by β (1→4) glycosidic linkages [8]. A biocompatible and biodegradable polysaccharide that can offer a non-protein matrix for tissue formation, is regarded as a supreme biomedical material. However, its applications are limited due to its lack of superior mechanical characteristics. To circumvent this limitation, CHI has been mixed with a variety of polymers and fillers, resulting in a wide range of CHI-based nanocomposites [8]. Important material characteristics that CHI possesses include biocompatibility, biodegradability, minimal immunogenicity, and bacteriostatic action [26]. CHI has been demonstrated to offer a non-protein matrix for tissue development, as well as the ability to drive cell proliferation and tissue organisation. Its chemical structure can be changed to increase the aforementioned properties as well as its solubility. The most often employed nanofillers in the production of CHI nanocomposites are: (i) layered silicates, such as clay; (ii) metal/ceramic nanoparticles; (iii) carbon nanotubes; and, more recently, (iv) graphene-based materials [8].

Alginate is a naturally occurring anionic polymer derived from brown seaweed that has been widely researched and utilised for various biomedical applications because of its biocompatibility, low toxicity, low cost, and moderate gelation caused by the addition of divalent cations such as Ca2+. Alginate gels have shown promise in bone regeneration through the administration of osteoinductive factors, bone-forming cells, or a combination of the two. These gels have an advantage over other materials for bone and cartilage regeneration because they can be introduced into the body in a minimally invasive manner, they can fill irregularly shaped defects, and they are easy to chemically modify with adhesion ligands and controlled release of tissue induction factors. However, alginate gels lack the mechanical characteristics to support load in the early phases of regeneration without fixing. They are also not degradable in physiological settings and stress the need of controlling their breakdown in order to avoid residual gels interfering with regeneration [27]. Limited studies on alginate are conducted, and limited literature is available.

Pure biomedical polymers have several advantages. These polymers may be used to create exact micro and nanoscale environments that promote cell adhesion, proliferation, and differentiation. They can also be customised for controlled medication delivery. Because of these benefits, they are being widely developed for tissue regeneration. Due to inherent flaws, pure biomedical polymers cannot imitate the mechanical properties of native tissues, particularly strength, elasticity, and modulus. Bioactive ceramic-based nanophases (bioactive glass and calcium phosphate) and various polymers (natural and synthetic polymers) have been hybridized to increase their mechanical and osteogenic capabilities [28].

Metals are also being increasingly used in bone tissue engineering due to their various advantages. Titanium is usually used as it is biocompatible, has high strength and rigidity, low density and weight, is thermophilic, and is resistant to corrosion [29]. The utilization of titanium was inspired by a successful outcome of using a titanium mesh for the reconstruction of maxillofacial defects. Titanium is a material that is extensively used in dentistry, craniomaxillofacial surgery, and orthopaedics. Occlusive titanium and microperforated titanium membranes have also been developed and are being utilised to treat peri-implant bone deficiencies for ridge augmentation. The biocompatibility and tissue performance similarities and differences between commercially pure titanium and titanium alloys have recently been examined. A cobalt-chromium (CoCr) alloy has also been proposed for guided bone regeneration (GBR). Despite the fact that this alloy is less biocompatible than titanium and titanium alloy, it has greater mechanical qualities (e.g., stiffness and toughness) [15]. Titanium oxide tubes are also used as nanomaterials in BTE.

C. Synthetic Polymers

These materials' thermo-modifiable characteristics allow them to produce excellent outcomes in BTE. They are often constructed of polyesters such as polyglycolic acid, polylactic acid, polycaprolactone, and copolymers in BTE [5]. Expanded polytetrafluoroethylene (e-PTFE) was the first synthetic polymer reported to be utilised for GBR; it is regarded as one of the most inert, stable polymers in the biological system [15]. It does not cause immunological reactions since it is resistant to being broken down by host tissues [30]. The structural integrity and tissue-exclusion function of the membrane are preserved by the chemical stability of e-PTFE.

Polylactic acid (PLA), polyglycolic acid (PGA), polycaprolactone (PCL), polyhydroxyl valeric acid, polyhydroxyl butyric acid, and their copolymers are examples of aliphatic polyesters that have been employed in the manufacture of GBR membranes [15]. The main advantages of such types of polymers are their manageability, processing, tuned biodegradability, and ability to encapsulate drugs [31,32]. Nevertheless, their degradation may give a strong inflammatory action, resulting in the resorption of the regenerative bone. Their insufficient rigidity and stability can be considered huge disadvantages [5]. The fast breakdown rate of aliphatic polyesters limits the barrier membrane's available function time and space-making ability, which may alter the result of bone regeneration. Nonetheless, studies have shown that polyester-based membranes can successfully preserve and enhance alveolar bone following tooth loss. The resorption rate of these membranes is mostly determined by the polymer utilised; PCL, for example, has stronger hydrophobicity and lower water solubility than PLA or PGA. Furthermore, copolymer-based membranes (e.g., lactide, caprolactone, glycolide, and trimethylene carbonate) have been proposed to minimise the resorption rate [15].

Few ceramic-based scaffolds are also emerging as new materials for GBR/BTE, for example, calcium sulfate (CaS) co-polymers and hydroxyapatite (HA)‐based membrane. CaS is a substance that is biocompatible, osteoconductive, and bioresorbable. It occurs naturally and may also be created through various synthetic processes. CaS-based membranes are created by hydrating CaS hemihydrate powder (plaster of Paris), resulting in a paste that can be moulded and hardened to a stiff material with relatively stable and less resorbable crystals [15].

HA is the most common calcium phosphate substance utilised in bone applications as it is less resorbable than any other CaS compound [15]. It is bioactive, biocompatible, nontoxic and osteoconductive, but has critical mechanical qualities like being brittle and unable to hold the heavy compressive load. HA also lacks osteoinductivity and true bone regeneration capabilities [33]. To improve their biological performance in vivo, the HA powder used to make pure ceramic membranes or other types of membranes has been mixed with bioactive ions such as strontium, silver, and zinc. Other ceramic materials, such as beta-tricalcium phosphate (β-TCP), have been used in resorbable membranes and have shown pro-osteogenic properties both in vitro and in vivo. Furthermore, adding bioactive glass nanoparticles to bioresorbable membranes has been proven in vitro to increase cell metabolic activity and mineralization [15]. β-TCP is proven to be biocompatible, osteoconductive, safe, and predictable, based on several preclinical and clinical studies. These materials show great potential for the generation of scaffolds effective in carrying and modulating the behaviour of MSCs [34]. β-TCP is used for the regeneration of hard tissue owing to its composition, biocompatibility, degradation, and regenerative ability [30]. However, easy fatiguability and fragility limit its application as a load-bearing polymer [31].

Discussion

To outline and then manufacture an effective bone graft, researchers and orthopaedics must have a thorough understanding of graft features such as osteogenesis, osteoinductivity, and osteoconductivity, as well as their various benefits and drawbacks. None of the grafts used have all of the desired attributes, such as biological safety, low donor morbidity, no size restrictions, long shelf life, low cost, and osteogenic, osteoinductive, osteoconductive, and angiogenic capabilities [4]. Concerns have been expressed about the possible dangers of nanoparticles to human health and the environment if they are exposed during their lifetime. Nanotoxicology, the science of manmade nanostructures that deal with health issues or adverse effects on living creatures, has recently received a surplus amount of interest. Understanding the molecular mechanisms of cell-nano biomaterials interactions is crucial [6]. Nanocomposite biomaterials are a novel class of materials that combine a biopolymeric and biodegradable matrix structure with bioactive and readily resorbable nano-sized fillers [35].

Three-dimensional printing (3DP) is a rapid prototyping technique that uses an inkjet printer to print a liquid binder onto powder biomaterials to create complex 3D structures for tissue engineering scaffolds. Direct fabrication of scaffolds from 3DP, on the other hand, limits material options due to manufacturing processes [4]. Advances in tissue engineering will greatly impact bone healing and regeneration in the near future. Perhaps polytherapy, which combines scaffolds, healing promoters, and stem cells with recent breakthroughs in three- and four-dimensional printing of tissue-engineered constructions, can overcome the current constraints in treating bone lesions [4].

## Conclusions

One of the most remarkable management modalities for crucial bone abnormalities is bone grafting. Autografts are still being considered a gold standard modality. Tissue-engineering-based grafts, allografts, and xenografts all have their limitations, for which new treatments such as gene therapy, polytherapy with scaffolds, healing promotive factors, and stem cells are being researched and developed. However, there are still problematic conditions and complexities that demand further advancements in bone regeneration membranes. Such membranes are thought to have bone-promoting, soft tissue compatibility, and antimicrobial capabilities. Guided bone regeneration membrane development has been mostly driven by the desired barrier function, user comfort, and clinical handling in various clinical circumstances, rather than a systematic attempt to improve biological results. However, a large body of experimental evidence shows that modifying the physicochemical and mechanical characteristics of membranes may accelerate bone regeneration. Unfortunately, numerous membranes have been marketed for clinical use but lack sufficient material characterization. The use of nanocomposites in bone regeneration is proving to be a really fruitful endeavour in orthopaedics. Even though further studies are still required to develop a flawless nanocomposite, the use of currently available nanocomposites is also proving to be highly beneficial.

Finally, 3DP is still in the initial and nascent stages of development, but it could provide new approaches and various attractive options in the upcoming future. The use of nanocomposites in regeneration provides a new field of research and intervention, which can be highly beneficial as a modality in various fields of medicine. Studies are still being carried out to limit the disadvantages of using technologies in the healthcare industry, with nanomaterials opening up new possibilities.
